# Poor prognostic factors of pharmacokinetic origin predict outcomes in inflammatory bowel disease patients treated with anti-tumor necrosis factor-α

**DOI:** 10.3389/fimmu.2024.1342477

**Published:** 2024-01-23

**Authors:** Elizabeth A. Spencer, Marla C. Dubinsky, Michael A. Kamm, Maria Chaparro, Paolo Gionchetti, Fernando Rizzello, Javier P. Gisbert, Emily K. Wright, Julien D. Schulberg, Amy L. Hamilton, Dermot P. B. McGovern, Thierry Dervieux

**Affiliations:** ^1^ Division of Gastroenterology, Icahn School of Medicine Mount Sinai, New York, NY, United States; ^2^ St Vincent’s Hospital and The University of Melbourne, Melbourne, VIC, Australia; ^3^ Hospital Universitario de La Princesa, Instituto de Investigación Sanitaria Princesa (IIS-Princesa), Universidad Autónoma de Madrid (UAM) and Centro de Investigación Biomédica en Red de Enfermedades Hepáticas y Digestivas (CIBEREHD), Madrid, Spain; ^4^ IRCCS Azienda Ospedaliero-Universitaria di Bologna Italy, Bologna, Italy; ^5^ DIMEC University of Bologna-Italy , Bologna, Italy; ^6^ F. Widjaja Inflammatory Bowel Institute, Cedars-Sinai Medical Center, Los Angeles, CA, United States; ^7^ Research and Development, Prometheus Laboratories, San Diego, CA, United States

**Keywords:** drug response, tumor necrosis factor, clearance, inflammatory bowel disease, pharmacogenetic

## Abstract

**Introduction:**

We evaluated baseline Clearance of anti-tumor necrosis factors and human leukocyte antigen variant (HLA DQA1*05) in combination as poor prognostic factors (PPF) of pharmacokinetic (PK) origin impacting immune response (formation of antidrug antibodies) and disease control of inflammatory bowel disease (IBD) patients treated with infliximab or adalimumab.

**Methods:**

Baseline Clearance was estimated in IBD patients before starting treatment using weight and serum albumin concentrations. HLA DQA1*05 carrier status (rs2097432 A/G or G/G variant) was measured using real time polymerase chain reaction. The outcomes consisted of immune response, clinical and biochemical remission (C-reactive protein<3 mg/L in the absence of symptoms), and endoscopic remission (SES-CD<3). Statistical analysis consisted of logistic regression and nonlinear mixed effect models.

**Results and discussion:**

In 415 patients enrolled from 4 different cohorts (median age 27 [IQR: 15-43] years, 46% females), Clearance>0.326 L/day and HLA DQA1*05 carrier status were 2-fold more likely to have antidrug antibodies (OR=2.3, 95%CI: 1.7-3.4; p<0.001, and OR=1.9, 95%CI: 1.4-2.8; p<0.001, respectively). Overall, each incremental PPF of PK origin resulted in a 2-fold (OR=2.16, 95%CI: 1.7-2.7; p<0.01) higher likelihood of antidrug antibody formation. The presence of both PPF of PK origin resulted in higher rates of antidrug antibodies (p<0.01) and lower clinical and biochemical remission (p<0.01). Each incremental increase in PPF of PK origin associated with lower likelihood of endoscopic remission (OR=0.4, 95%CI: 0.2-0.7; p<0.001). Prior biologic experience heightened the negative impact of PPF of PK origin on clinical and biochemical remission (p<0.01). Implementation of proactive therapeutic drug monitoring reduced it, particularly during maintenance and in the presence of higher drug concentrations (p<0.001). We conclude that PPF of PK origin, including both higher Clearance and carriage of HLA DQA1*05, impact outcomes in patients with IBD.

## 1 Introduction

Predicting response to monoclonal antibody therapies remains an unmet need in the management of immune-mediated inflammatory disease, particularly in inflammatory bowel disease (IBD; Crohn’s disease [CD] and ulcerative colitis [UC]), that causes progressive intestinal damage, which impacts the quality of life of affected patients (1). After two decades of intensive research, it is clear that the response to anti-tumor necrosis factor-α; (TNF) such as infliximab (IFX) and adalimumab (ADA) is complex and pathway dependent (2, 3). Undoubtedly, the response to anti-TNF is also a function of suboptimal pharmacokinetics (PK) (4) where immune response and formation of neutralizing antibodies preclude the achievement of the minimally effective concentration required for disease control (5). In fact, the prediction of suboptimal baseline PK in patients starting induction is likely to be important, as a countermeasure of simple dose intensification may prevent the potential negative impact of lower concentration on disease control in a susceptible individual.

One of the most promising genetic markers associated with the immune response to anti-TNF and formation of antibodies to IFX (ATI) or ADA (ATA) is HLA DQA1*05 (tagged rs2097432 A/G), and substantial evidence supports the value of the genotype as recently reported in a meta-analysis (6) with 75% higher risk of immunogenicity compared with non-carriers and twofold higher risk of secondary loss of response. The precise mechanism of action is well established and combines the recognition of the antigenic proteolytic fragments of the monoclonal antibody itself by the immune system and clonal expansion to produce neutralizing antibodies. However, HLA DQA1*05 has modest performances when associated with PK and pharmacodynamic outcomes, illustrating the complexity of a low penetrance single variant with outcome.

Recently, several reports have established that baseline clearance calculated from covariates estimated in the population PK model is associated with outcome in IBD (7, 8), where higher clearance reflects intrinsic suboptimal PK (e.g., recirculation of the IgG through the neonatal receptor and/or higher weight) as well as an individual’s inflammatory burden, which consumes the drug. This unfavorable state is only worsened in the presence of the HLA DQA1*05 carrier status and the associated immune response.

In this report, we evaluate accelerated baseline clearance and HLA DQA1*05 carrier status as PPFs of PK origin impacting immune response and therapeutic outcomes in IBD. Our results show a significant impact of higher clearance and presence of HLA DQA1*05 carrier status on immune response where the cumulative presence of both PPFs of PK origin is associated with a high likelihood of treatment failure.

## 2 Methods

### 2.1 Patients

Patients with IBD were enrolled from four different cohorts starting subcutaneous ADA or intravenous IFX treatment (9–12). Internal review boards approved the studies, and patient informed consent was collected. The first cohort (BOLOGNA) was performed in the context of a 1-year prospective observational clinical trial aimed at identifying biomarkers and predictors of a failure to respond to ADA in patients with CD (11). The second cohort (PREDICROHN) was a prospective multicenter cohort study in patients with CD naïve to biologics with active luminal disease (12); participants were started on ADA and IFX and followed up longitudinally. The third cohort (STRIDENT) was from an open-label, single-center, randomized controlled trial evaluating dose intensity in participants with symptomatic intestinal Crohn’s disease strictures (9). The patients in the fourth cohort (Proactive dosing cohort, PRECISION IFX trial [NCT02624037]) received proactive dose intensification using therapeutic drug monitoring (TDM) and iDose dashboard (Baysient, LLC, Fort Myers, FL, USA) to target therapeutic concentrations above 17 µg/mL and 10 µg/mL during induction and maintenance, respectively (10); the impact of proactive TDM in preventing immunization to IFX has been reported elsewhere (13). Patients from each cohort were followed up longitudinally at each visit during their maintenance treatment. Blood specimens were collected periodically during maintenance and always at the trough for IFX; sera were isolated and stored at subzero temperature (−80°C) until analysis.

### 2.2 Pharmacokinetic and pharmacogenetic measurements

Serum ADA and IFX concentrations and their respective antibodies (ATA and ATI, respectively) were determined using drug-tolerant homogenous mobility shift assay in the clinical laboratory at Prometheus Laboratories (San Diego, CA, USA) (14, 15). All specimens were collected in serum separator tubes. Pre-analytical experiments have shown that the analytes are stable for at least 14 days at room temperature in serum. Serum was stored at −80°C within 72 hours of isolation. The lower and upper limits of quantification of the drug assay were 1.6 µg/mL and 50 µg/mL, respectively, for ADA and 0.8 µg/mL and 34 µg/mL, respectively, for IFX. The cutoffs associated with ATA and ATI status (corresponding to the 97.5th percentile of normal health) were 1.7 U/mL and 3.1 U/mL, respectively. Serum albumin and C-reactive protein (CRP) were determined using immunochemistry (IMMAGE 800 Protein Chemistry Analyzer, Beckman Coulter, Brea, CA, USA) in the clinical laboratory at Prometheus Laboratories). Carriage of the HLA-DQA1*05 (presence of rs2097432 AG or GG variant) was determined from genomic DNA extracted from serum or whole blood using real-time PCR with allelic discrimination (13).

### 2.3 Derivatization of baseline clearance

The population PK parameters were estimated from the BOLOGNA and PREDICROHN cohorts (113 patients who received ADA and 553 samples/observations) using non-linear mixed-effects modeling (one compartment with linear elimination), with random effects on apparent clearance (CL/F) with albumin levels and weight as covariates. The covariate estimates of weight and albumin from the population PK model were applied to calculate baseline clearance for ADA and IFX in all patients before starting treatment. Cutoff for higher clearance corresponds to the typical value determined from that population PK model and was applied unmodified to all other cohorts receiving ADA or IFX. This clearance calculation was used for both IFX and ADA baseline clearance.

### 2.4 Outcome variables and statistical analysis

Immune response to ADA and IFX consisted of antidrug antibody formation (above cutoff) during induction and maintenance, anytime (corresponding to immune response detected at any of the time points where serum was available for PK analysis), and at all cycles (corresponding to immune response detected at all of the time points where serum was available for PK analysis). The clinical outcome determined at each study visit was CRP-based clinical remission status, defined as CRP levels below 3 mg/L in the presence of clinical remission (Crohn’s Disease Activity Index<150 points or Harvey–Bradshaw index (HBI) below 5 points for CD, or partial Mayo below 2 points for UC).

Endoscopic remission (ER) was available in CD only and corresponded to the Simple Endoscopic Score for CD (SES-CD< 3 points) available during treatment in the BOLOGNA and STRIDENT cohorts. Statistical analysis consisted of logistic regression with odds ratio (OR; with 95% confidence interval). Results were expressed as median with interquartile ranges (IQRs), as appropriate.

The impact of baseline clearance and HLA DQA1*05 carrier status on outcomes was estimated using longitudinal repeated event analysis using non-linear mixed-effects modeling via Monolix (Lixoft, 2021R2). Prior biologic exposure and implementation of proactive TDM (from the PRECISION cohort) were used as covariates in the analysis, as appropriate. Logistic regression and Mann–Whitney testing were used as appropriate.

## 3 Results

A total of 415 patients (median age 27 [IQR: 15–43] years, 46% female) were enrolled in the study (n = 185 ADA and n = 230 IFX). PK measurements and CRP-based clinical remission status during maintenance were available in a total of 1,893 cycles collected with PK specimens; antidrug antibodies were detected in 15% of patients at any time point. Results are presented in **Table 1**. There was no difference in concentrations between IFX and ADA during maintenance (IFX: median of 9.9 µg/mL [IQR 5.0–15.1 µg/mL]; ADA: median of 10.7 µg/mL [IQR 6.9–14.6 µg/mL]; p > 0.6).

**Table 1:** Patient characteristics.

	BOLOGNA (Italy)	PREDICROHN (Spain)	STRIDENT (Australia)	PRECISION (USA)	All cohorts
Anti-TNFs	ADA	ADA/IFX	ADA	IFX	ADA/IFX
Crohn’s disease	100% (53/53)	100% (112/112)	100% (77/77)	73% (125/173)	88%% (367/415)
Prior biologics	30% (16/53)	0% (0/112)	12% (9/77)	22% (38/173)	15% (63/415)
Age (years)	24 (26; 44)	39 (29; 50)	44 (30; 52)	15 (12; 17)	27 (15; 43)
Gender (female)	36% (19/53)	48% (54/112)	51% (39/77)	46% (79/173)	46% (191/415)
Weight at baseline	67 (60; 78)	64 (57; 76)	78 (66; 87)	45 (33; 59)	61 (46; 73)
ALB at baseline	3.9 (3.8; 4.2)	3.8 (3.3; 4.4)	3.7 (3.5; 3.9)	3.2 (2.8; 3.7)	3.6 (3.0; 4.0)
HLA DQA1*05 carriage	47% (25/53)	37% (42/112)	27% (21/77)	46% (80/173)	40% (168/415)
CL > 0.326 L/day	51% (27/53)	49% (55/112)	79% (61/77)	42% (72/173)	52% (215/415)
Responder score >0	79% (42/53)	67% (75/112)	86% (66/77)	69% (120/173)	73% (303/415)
Responder score >1	19% (10/53)	20% (22/112)	21% (16/77)	18% (32/173)	19% (80/415)
Antibodies (anytime)	23% (12/53)	16% (18/112)	14% (11/77)	13% (23/173)	15% (64/415)
Antibodies all cycles	15% (28/182)	11% (70/614)	8% (24/285)	4% (34/930)	8% (156/2011)
CRP-based clinical rem.	47% (28/182)	44% (235/535)	45% (113/250)	52% (487/930)	49% (919/1893)
SES-CD	1 (0; 4)	NA	3 (0; 8)	NA	3 (0; 6)
SES-CD ≥ 3	43% (39/90)	NA	64% (35/55)	NA	51% (74/145)
Maintenance cycles	3.4 (182)	4.5 (507)	2.7 (208)	2.5 (434)	3.2 (1331)
Trough concentrations	10 (5.2; 12.8)	8.0 (3.9; 12.2)	13.2 (8.2; 18.0)	12.3 (8.3; 18.0)	10.2 (6.0; 15.1)

Results are expressed as median IQR or % (n/N), as appropriate.

SES-CD, Simple Endoscopic Score for Crohn’s Disease.

NA, not available.

### 3.1 Baseline clearance and calculation of PF of PK origin score

Baseline clearance was calculated using the parameter estimates derived from the population PK model from ADA patients (BOLOGNA and PREDICROHN cohorts) as follows:


CL= (EXP(LOG(0.326) + 0.458∗LOG(WT/70) − 0.768∗LOG(ALB/4.0)),

where *WT* is weight in kg and *ALB* is serum albumin level in g/dL.

The PPF of PK origin score was calculated as the sum of higher clearance (>0.326 L/day, 1 point) and HLA DQA1*05 G carrier status (1 point) and ranged from 0 to 2 (0 corresponding to the absence of both PPFs of PK origin, 1 corresponding to either clearance > 0.326 L/day or HLA DQA1*05 G carrier status, and 2 corresponding to the presence of both PPFs of PK origin).

### 3.2 Impact of PPFs of PK origin on immune response during treatment

Longitudinal repeated event analysis over the treatment period revealed that longer time on therapy associated with immune response and antidrug antibody formation (θ_time_ = + 0.006 ± 0.001, p< 0.001), where the presence of PPFs of PK origin (clearance > 0.326 L/day; HLA DQA1*05 carrier status) at baseline each independently and significantly contributing to immunization during treatment (θ_Clearance>0.326_ = +2.2 ± 0.70, p = 0.02, and θ_HLA DQA1*05 carrier_ = +1.5 ± 0.70, p = 0.03, respectively) (θ_pop_ = −9.6 ± 1.0). Patients with clearance > 0.326 L/day and HLA DQA1*05 carrier status were twofold more likely to present with treatment cycles with antidrug antibodies (OR = 2.3, 95%CI: 1.7–3.4, p< 0.001, and OR = 1.9, 95%CI: 1.4–2.8, p< 0.001, respectively).

**Figure 1:**
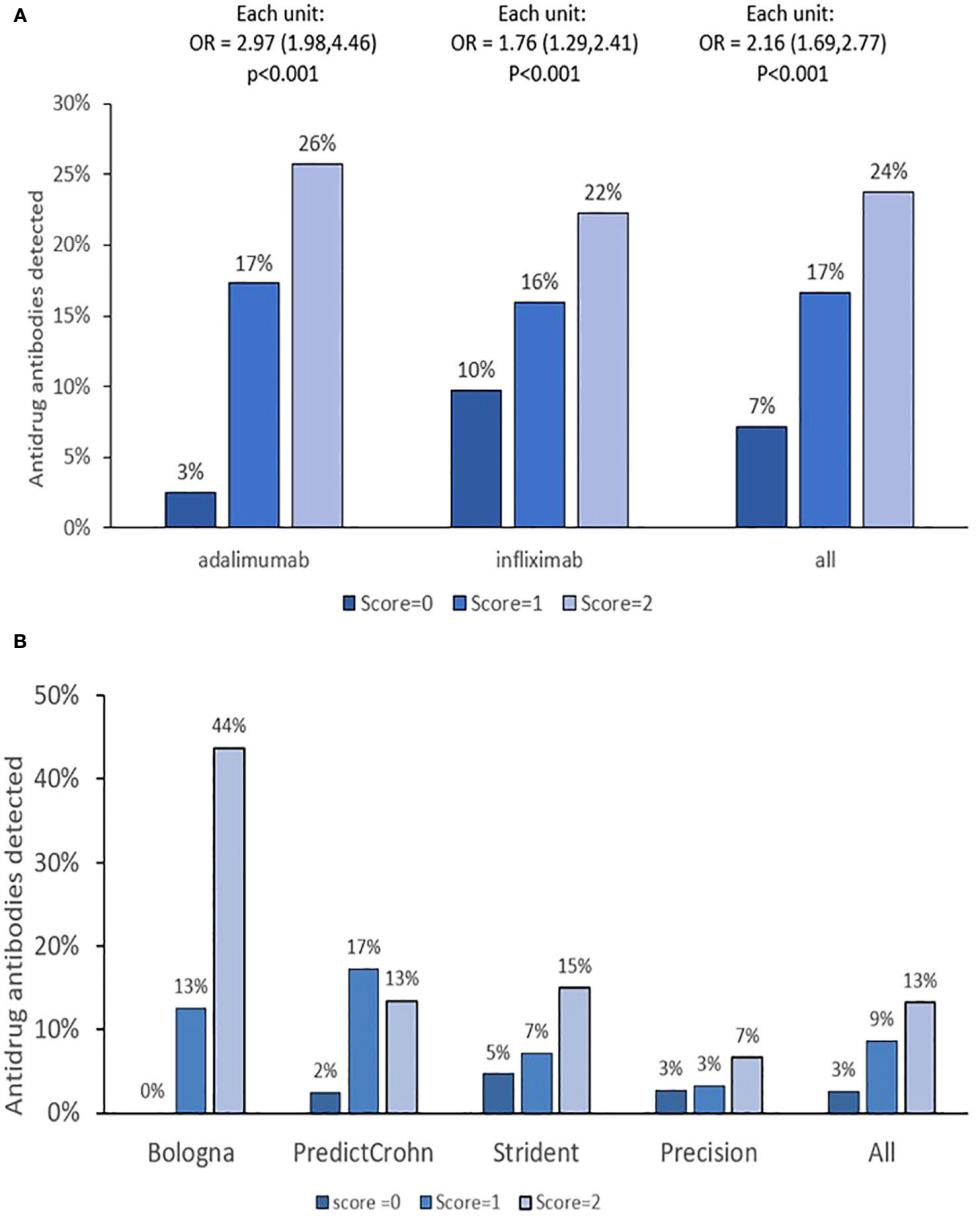
PPF of PK origin score and treatment cycles with antidrug antibodies. **(A, B)** The results by monoclonal antibody and cohorts, respectively. OR is given with 95% confidence intervals. Immune response (ATA or ATI) detected anytime during treatment was calculated. PPF, poor prognostic factor.

When combined, the increased number of PPFs of PK origin resulted in a higher risk of immune response for both ADA and IFX (**Table 2**). Results by cohort are presented in **Supplementary Table 2**. The incidence of antidrug antibodies by PPFs of PK origin is presented in **Figure 1**. Overall, each incremental PPF of PK origin resulted in a 2-fold (OR=2.16, 95%CI: 1.7-2.7; p<0.01) higher likelihood of antidrug antibody formation. Also, antidrug antibodies detected at the time of clinical assessment were associated with a lower likelihood of CRP-based clinical remission (OR = 0.5, 95%CI: 0.3–0.7, p< 0.001) and endoscopic remission (OR = 0.2, 95%CI: 0.1–0.7, p = 0.003). There was no impact of prior biologic treatment on immune response (data not shown).

**Table 2:** Poor prognostic factors of pharmacokinetic origin and immune response to anti-TNFs.

Parameter	Adalimumab	Infliximab	All cohorts
**θ_pop_**	−11.3 ± 2.9 (p< 0.001)	−9.1 ± 1.2 (p< 0.001)	−10.1 ± 1.2 (p< 0.001)
**θ_cov_: score =1 versus 0**	+4.5 ± 2.5 (p = 0.07)	+1.8 ± 1.1 (p = 0.102)	+2.5 ± 0.9 (p = 0.006)
**θ_cov_: score =2 versus 0**	+6.2 ± 2.6 (p = 0.017)	+2.6 ± 1.2 (p = 0.030)	+3.8 ± 1.1 (p< 0.001)
**θ_time_**	+0.006 ± 0.001 (p = 0.030)	+0.006 ± 0.001 (p = 0.030)	+0.006 ± 0.001 (p< 0.001)

Model, logit(Probability of CRP Based Remission) = θ_pop_ + θ_covi_ * cov_i_ + ···.

### 3.3 Impact of PPFs of PK origin on CRP-based clinical remission

Longitudinal repeated event analysis over the treatment period revealed that longer time on therapy was associated with a higher probability of achieving CRP-based clinical remission (θ_time_ = +0.004 ± 0.001, p< 0.001). Additionally, higher baseline clearance (>0.326 L/day, θ_Clearance>0.326_ = −1.1 ± 0.2, p< 0.001) and prior biologic therapy (θ_prior_ biologics = −1.2 ± 0.3, p< 0.001) negatively impacted achievement of CRP-based clinical remission, an effect that was modified by proactive TDM (θ_proactive_ tdm = 0.6 ± 0.2, p< 0.003) but not HLA DQA1*05 carrier status (θ_HLA DQA1*05 carrier_ = 0.1 ± 0.2, p = 0.62) (θ_pop_ = −0.5 ± 0.2, p = 0.012).

**Figure 2:**
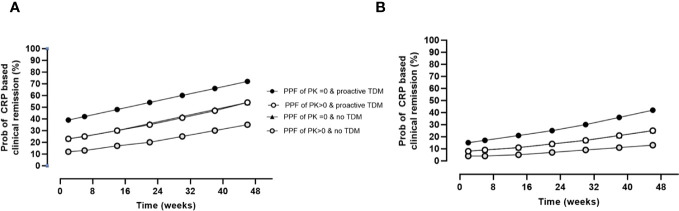
Impact of PPFs of PK origin by proactive TDM and prior biologics on CRP-based clinical remission status over time. Estimates are as follows: θ_po_p = 0.5 ± 0.3 (p = 0.100); θ_cov_, prior biologics = −1.2 ± 0.3 (p< 0.001); θ_cov_, PPFs of PK origin >0 = −0.8 ± 0.3 (p = 0.008); θ_cov_, proactive TDM = 0.8 ± 0.2 (p< 0.001); θ_Time_ = 0.004 ± 0.001 (p< 0.001). **(A)** Probability of CRP-based clinical remission in patients naïve to biologics. **(B)** Probability of CRP-based clinical remission in patients with prior biologics. The probability of CRP-based remission in the presence of proactive TDM and PPK > 0 was indistinguishable from the probability from the probability of with no TDM and PPF of PK = 0. PPF, poor prognostic factor; PK, pharmacokinetic; TDM, therapeutic drug monitoring; CRP, C-reactive protein.

The impact of the PPF of PK score (>0) on CRP-based clinical remission status adjusting for prior biologics, proactive TDM, and time on therapy is presented in **Figure 2**. The analysis revealed a significant impact of prior biologics on worse outcomes, while proactive TDM was associated with improved disease control, an effect that was also dependent on the presence of PF of PK origin. Results by cohorts and biologics are presented in **Supplementary Tables 3**, **4**, respectively.

**Figure 3:**
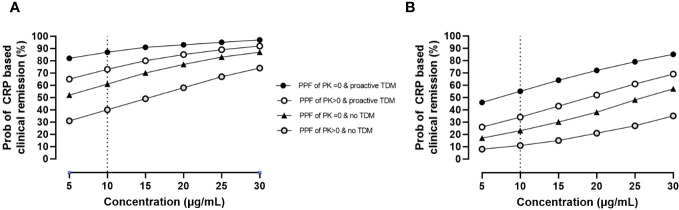
Impact of PPFs of PK origin by proactive TDM and prior biologics on CRP-based clinical remission status by exposure during maintenance. Estimates are as follows: θ_pop_ = 0.3 ± 0.4 (p = 0.453); θ_cov_, prior biologics = −1.7 ± 0.5 (p = 0.001); θ_cov_, PPFs of PK origin >0 = −0.9 ± 0.4 (p = 0.024); θ_cov_, proactive TDM = 1.4 ± 0.3 (p< 0.001); θ_concentrations_ = 0.07 ± 0.01 (p< 0.001). **(A)** Probability of CRP-based clinical remission in patients naïve to biologics. **(B)** Probability of CRP-based clinical remission in patients with prior biologics. PPF, poor prognostic factor; PK, pharmacokinetic; TDM, therapeutic drug monitoring; CRP, C-reactive protein.

During maintenance, longitudinal analysis also revealed that prior biologics, proactive TDM, and the presence of at least one PPF of PK origin impacted therapeutic outcomes with higher concentrations resulting in improved disease control. Results are presented in **Figure 3**.

### 3.4 Impact of PPFs of PK origin on endoscopic outcomes

**Figure 4:**
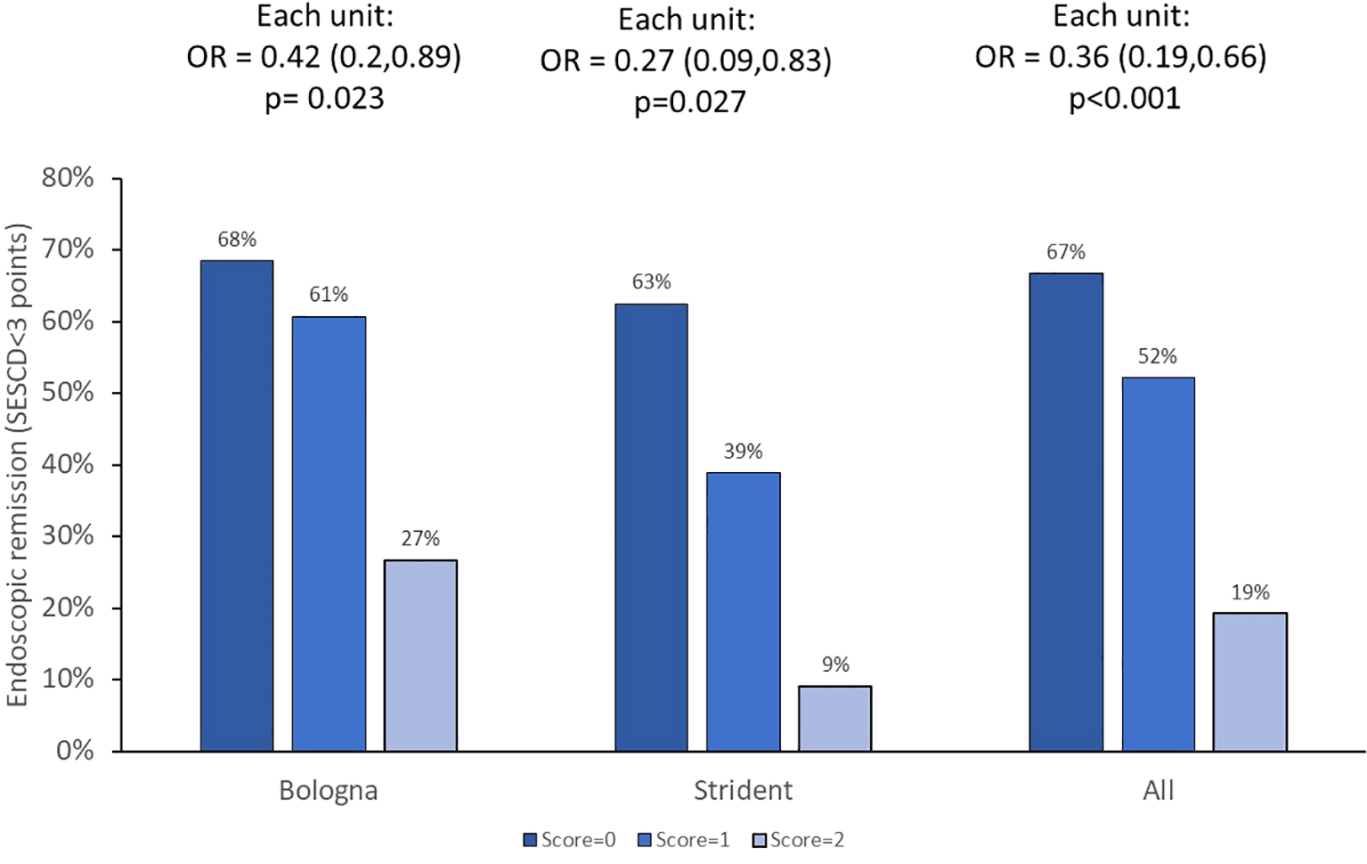
Endoscopic remission and PPFs of PK origin. The OR with 95%CI for each incremental unit of the PPF of PK origin is given. PPF, poor prognostic factor; PK, pharmacokinetic.

Endoscopic outcomes (n = 145 assessments) were available in ADA-treated patients enrolled in the BOLOGNA and STRIDENT cohorts. ATA status detected anytime during treatment was associated with a 0.2-fold (95%CI 0.1–0.7) (p = 0.002) lower likelihood of having endoscopic remission. Multivariate logistic regression analysis revealed that higher clearance (adjusted OR = 0.3, 95%CI: 0.2–0.7, p = 0.004) and presence of HLA DQA1*05 carriage (adjusted OR = 0.4, 95%CI: 0.2–0.8, p = 0.013) independently and significantly impacted endoscopic remission. Cumulatively, each incremental increase in PPFs of PK origin resulted in a lower likelihood of endoscopic remission (OR = 0.4, 95%CI: 0.2–0.7, p< 0.001). Results are presented in **Figure 4**. There was no association between prior biologics on worse endoscopic outcomes (data not shown).

## 4 Discussion

In this report, we have established that the PPFs of PK origin, both higher baseline clearance of IFX and ADA and carriage of HLA DQA1*05, impact immune response and disease control in patients with IBD. These PPFs of PK origin specifically detect a signature associated with suboptimal PK during treatment with anti-TNF-α;. The first PPF of PK origin corresponds to accelerated clearance of the monoclonal antibody itself and is representative of its baseline intrinsic recirculation (with albumin as a proxy) (16) and also the inflammatory burden that accelerates the consumption of the drug from the central compartment (17). The second PPF of PK origin corresponds to the HLA DQA1*05 variant (detected as the A/G and G/G genotype), which informs on the immunization risk by presentation of the monoclonal antibody for clonal expansion and production of antidrug antibodies (6).

Our finding that the combination of these two PPFs of PK origin resulted in worse outcomes was expected. Notably, in addition to increasing immune response and antidrug antibodies, disease control was also worsened with reductions in clinical remission across all four cohorts. Our analysis further revealed that the detrimental effects of the PPFs of PK origin on CRP-based clinical remission were heightened in those who were biologic-experienced and lessened in those utilizing proactive TDM, as seen in the PRECISION cohort (10). With regard to endoscopic outcomes available from two separate cohorts, suboptimal PK secondary to the PPFs of PK origin resulted in worse outcomes with no impact of prior biologics.

Our data have some strengths and limitations in this population of IBD patients who all started an anti-TNF. The strengths include multiple, rich cohorts enrolled, the availability of endoscopic outcomes (at least with ADA) and availability of PK outcomes, and the fact that the responder score and clearance derived from the ADA-treated cohorts (BOLOGNA and PREDICROHN) also generally replicated in the IFX cohorts and the STRIDENT ADA cohorts. Limitations arise from the retrospective nature of our study, and it will be important to prospectively evaluate the value of the PPFs of PK origin.

There are potential direct clinical applications of these findings. The measurement and presence of the PPFs of PK origin before starting treatment could inform providers on the appropriateness of drug selection and dose intensification strategies to achieve exposure commensurate with disease control and thus remediate any suboptimal PK. Results may also identify which patients may benefit from combination therapy with a thiopurine to decrease the formation of antidrug antibodies, allowing for more sophisticated therapeutic decision-making and limiting unnecessary risk of adverse events in patients where there is an absence of PPFs of PK origin (18).

In conclusion, a serogenetic panel combining higher baseline clearance and HLA DQA1*05 is associated with outcomes in patients with IBD treated with anti-TNF therapies. Whether these PPFs of PK origin are also associated with outcomes in other immune-mediated inflammatory diseases is not known, but we hypothesize that their presence resulting in suboptimal PK is likely to result in lesser disease control as well, at least among the group of patients with active disease and high inflammatory burden.

## Data availability statement

The original contributions presented in the study are included in the article/**Supplementary Material**, further inquiries can be directed to the corresponding author/s.

## Ethics statement

The studies involving humans were approved by Mount Sinai Medical Center, St Vincent Hospital, University de la Princessa, University de Bologna. The studies were conducted in accordance with the local legislation and institutional requirements. Written informed consent for participation in this study was provided by the participants’ legal guardians/next of kin.

## Author contributions

ES: Conceptualization, Data curation, Formal analysis, Methodology, Writing – review & editing. MD: Conceptualization, Data curation, Formal analysis, Writing – review & editing, Funding acquisition, Investigation, Project administration, Supervision. MK: Conceptualization, Data curation, Project administration, Supervision, Writing – review & editing. MC: Data curation, Project administration, Supervision, Writing – review & editing, Conceptualization. PG: Data curation, Project administration, Conceptualization, Writing – review & editing. FR: Writing – review & editing, Conceptualization, Data curation. JG: Conceptualization, Data curation, Writing – review & editing, Project administration. EW: Conceptualization, Data curation, Writing – review & editing, Methodology. JS: Conceptualization, Data curation, Writing – review & editing. AH: Conceptualization, Data curation, Writing – review & editing. DM: Conceptualization, Writing – review & editing, Investigation, Validation. TD: Conceptualization, Data curation, Formal analysis, Investigation, Methodology, Project administration, Validation, Writing – original draft.
