# Corrigendum: Poor prognostic factors of pharmacokinetic origin predict outcomes in inflammatory bowel disease patients treated with anti-tumor necrosis factor-α

**DOI:** 10.3389/fimmu.2024.1383704

**Published:** 2024-02-27

**Authors:** Elizabeth A. Spencer, Marla C. Dubinsky, Michael A. Kamm, Maria Chaparro, Paolo Gionchetti, Fernando Rizzello, Javier P. Gisbert, Emily K. Wright, Julien D. Schulberg, Amy L. Hamilton, Dermot P. B. McGovern, Thierry Dervieux

**Affiliations:** ^1^ Division of Gastroenterology, Icahn School of Medicine Mount Sinai, New York, NY, United States; ^2^ St Vincent’s Hospital and The University of Melbourne, Melbourne, VIC, Australia; ^3^ Hospital Universitario de La Princesa, Instituto de Investigación Sanitaria Princesa (IIS-Princesa), Universidad Autónoma de Madrid (UAM) and Centro de Investigación Biomédica en Red de Enfermedades Hepáticas y Digestivas (CIBEREHD), Madrid, Spain; ^4^ IRCCS Azienda Ospedaliero-Universitaria di Bologna Italy, Bologna, Italy; ^5^ DIMEC University of Bologna-Italy, Bologna, Italy; ^6^ F. Widjaja Inflammatory Bowel Institute, Cedars-Sinai Medical Center, Los Angeles, CA, United States; ^7^ Research and Development, Prometheus Laboratories, San Diego, CA, United States

**Keywords:** drug response, tumor necrosis factor, clearance, inflammatory bowel disease, pharmacogenetic

In the published article, there was an error. In **Table 1**, the percentage of HLA DQA1*05 carriage for “all cohort” was incorrect and was corrected to 40% (168/415).

In the Abstract, and Results, section 3.2. *Impact of PPF of PK origin on Immune response during treatment*, there was an error in the p value reported: “Overall, each incremental PPF of PK origin resulted in a 2-fold (OR=2.16, 95%CI: 1.7-2.7; p<0.11) higher likelihood of antidrug antibody formation”.

The corrected sentence appears below:

“Overall, each incremental PPF of PK origin resulted in a 2-fold (OR=2.16, 95%CI: 1.7-2.7; p<0.01) higher likelihood of antidrug antibody formation”.

The authors apologize for these errors and state that this does not change the scientific conclusions of the article in any way. The original article has been updated.

