# Supplementary material for: Poor prognostic factors of pharmacokinetic origin predict outcomes in inflammatory bowel disease patients treated with anti-tumor necrosis factor-α
Source: Front Immunol. 2024 Jan 23;15:1342477. doi: 10.3389/fimmu.2024.1342477 (PMC10929708; doi:10.3389/fimmu.2024.1342477)
Supplement: Supplementary file 1 [file DataSheet_1.docx]

Supplementary Materials

POOR PROGNOSTIC FACTORS OF PHARMACOKINETIC ORIGIN PREDICT OUTCOMES IN INFLAMMATORY BOWEL DISEASE PATIENTS TREATED WITH ANTI-TUMOR NECROSIS FACTOR-α

**Population PK model of Clearance during ADA treatment**

The population PK parameters were estimated from the first and the second cohort who received ADA (113 patients with 553 observations). Specifically, a one-compartment model with first-order absorption and elimination was used to describe ADA concentration versus time data. The model was fit to pharmacokinetic data via nonlinear mixed-effects modeling (Monolix 5.1.0; Lixoft, Antony, France) using the stochastic approximation expectation-maximization approach. The population parameters estimated included apparent clearance, CL/F (L/days) and apparent volume, V/F (L). Due to the limited sampling absorption (ka) was fixed to 0.2 (L/days) from the literature. The inter-individual and inter-occasion variability of the parameters was assumed to be log-normally distributed. ADA concentrations below the lower limit of quantification (1.6 µg/mL) were treated as censored and the M3 method for handling these values was used as implemented in Monolix. An additive residual error model was used with assumed normal distribution. The effects of the covariates weight and serum albumin were evaluated for influence on ADA pharmacokinetics. Parameter estimates are presented in **Table S1**.

**Table S1 parameter estimates**

| Parameter | Estimate | RSE (%) |
| --- | --- | --- |
| CLpop (L/day), apparent | 0.326 | 0.016 (5.08) |
| V pop (L), apparent | 6.28 | 0.493 (7.86) |
| Ka (day^-1^) | 0.2 | FIXED |
| Ω CL (IIV) | 0.414 | 0.065 (15.8) |
| beta WT | 0.458 | 0.219 (47.8) |
| beta ALB | -0.768 | 0.301 (39.2) |
| Additive error | 2.98 | 0.719 (15.8) |
| IOV | 0.296 | 0.095 (32.1) |

RSE: Relative Standard Error Estimate; IIV: Inter-Individual Variability; IOV: Inter-Occasion Variability

**Table S2: PPF of PK origin and Immune response to anti-TNFs**

| parameter | Bologna | Predicrohn | Strident | Precision | All cohorts |
| --- | --- | --- | --- | --- | --- |
| θ_pop_ | -27.1±7.4  (p<0.001) | -18.5±4.6  (p<0.001) | -9.0±3.2  (p=0.004) | +5.2±0.7  (p<0.001) | **-10.1±1.2**  **(p<0.001)** |
| θ_cov_: score =1 versus 0 | +21.6±7.3  (p=0.003) | +7.9±3.5  (p=0.025) | +1.3±2.8  (p=0.646) | +0.4±0.7  (p=0.560) | **+2.5±0.9**  **(p=0.006)** |
| θ_cov_: score =2 versus 0 | +26.1±7.7  (p=0.001) | +6.3±3.7  (p=0.091) | +2.9±3.1  (p=0.354) | -1.3±0.8  (p=0.089) | **+3.8±1.1**  **(p<0.001)** |
| θ_time_ | +0.001±0.003  (p=0.886) | +0.012±0.003 (p<0.001) | +0.005±0.002 (p=0.047) | +0.002±0.001 (p=0.175) | **+0.006±0.001 (p<0.001)** |

*Model: logit(Probability of ATI or ATA)= θ_pop_+θ_covi_*cov_i_+…

**Table S3: PPF of PK origin and CRP based clinical remission status**

| parameter | Bologna | PREDICROHN | Strident | Precision | All cohorts |
| --- | --- | --- | --- | --- | --- |
| θ_pop_ | +1.8±1.6  (p=0.115) | +0.7±0.5  (p=0.115) | +0.9±1.0  (p=0.386) | -0.7±0.3  (p=0.021) | **-0.3±0.2**  **(p=0.305)** |
| θ_cov_: score =1 versus 0 | -1.1±1.4  (p=0.431) | -1.2±0.6  (p=0.033) | -2.4±1.1  (p=0.027) | -0.2±0.3  (p=0.624) | **-0.8±0.3**  **(p=0.007)** |
| θ_cov_: score =2 versus 0 | -4.0±2.0  (p=0.040) | -0.7±0.7  (p=0.339) | -1.0±1.1  (p=0.397) | -1.3±0.4  (p=0.002) | **-1.20±0.4**  **(p=0.001)** |
| θ_time_ | +0.004±0.004 (p=0.323) | +0.002±0.001 (p=0.054) | +0.003±0.001 (p=0.036) | +0.008±0.001 (p<0.001) | **+0.004±0.0005 (p<0.001)** |

*Model: logit(Probability of CRP Based Remission)= θ_pop_+θ_covi_*cov_i_+…

**Table S4: PPF of PK origin and CRP based clinical remission status**

| parameter | Adalimumab | Infliximab | All cohorts |
| --- | --- | --- | --- |
| θ_pop_ | 1.0±0.5  (p=0.05) | -0.7±0.5  (p=0.162) | -0.3±0.2  (p=0.305) |
| θ_cov_: score =1 versus 0 | -1.4±0.6  (p=0.02) | -0.3±0.3  (p=0.317) | -0.8±0.3  (p=0.007) |
| θ_cov_: score =2 versus 0 | -1.6±0.7  (p=0.02) | -0.8±0.4  (p=0.046) | -1.20±0.4  (p=0.001) |
| θ_time_ | +0.0007±0.0009 (p=0.43) | +0.006±0.0007 (p<0.001) | +0.004±0.0005 (p<0.001) |

*Model: logit(Probability of CRP Based Remission)= θ_pop_+θ_covi_*cov_i_+…
